# The post hoc analysis comparing the severity grades of chemoradiotherapy-induced oral mucositis scored between the central and local assessors in a multicenter, randomized controlled trial of rebamipide for head and neck cancer

**DOI:** 10.1007/s10147-018-1355-7

**Published:** 2018-11-13

**Authors:** Takao Ueno, Sadamoto Zenda, Tetsuhito Konishi, Takashi Yurikusa, Yoshiyuki Shibasaki, Hisashi Nagamoto, Masato Fujii

**Affiliations:** 1 Department of General Internal Medicine, Dentistry, Oncologic Emergency, National Cancer Center Hospital, 5-1-1, Tsukiji, Chuo-ku, Tokyo, 104-0045 Japan; 2 Division of Radiation Oncology and Particle Therapy, National Cancer Center Hospital East, 6-5-1 Kashiwa-no-ha, Kashiwa, Chiba 277-8577 Japan; 3 Innovation Center for Supportive, Palliative and Psychosocial Care, National Cancer Center Hospital, 5-1-1, Tsukiji, Chuo-ku, Tokyo, 104-0045 Japan; 4 Department of Dentistry, National Cancer Center Hospital East, 6-5-1 Kashiwa-no-ha, Kashiwa, Chiba 277-8577 Japan; 5 Division of Dentistry and Oral Surgery, Shizuoka Cancer Center, 1007 Shimonagakubo, Nagaizumi-cho, Sunto-gun, Shizuoka, 411-8777 Japan; 6 Medical Affairs, Otsuka Pharmaceutical Co., Ltd., Shinagawa Grad Central Tower 2-16-4, Konan, Minato-ku, Tokyo, 108-8242 Japan; 7 Medical Affairs, Otsuka Pharmaceutical Co., Ltd., 463-10 Kagasuno, Kawauchi-cho, Tokushima, 771-0192 Japan; 8 Department of Otolaryngology, Eiju General Hospital, 2-23-16 Higashiueno, Taito-ku, Tokyo, 110-8645 Japan

**Keywords:** Chemoradiotherapy-induced oral mucositis, Head and neck cancer, Post hoc analysis, Rebamipide liquid, Common Terminology Criteria for Adverse Events (CTCAE) version 3.0, Independent central review

## Abstract

**Background:**

In the treatment of head and neck cancer, severity of chemoradiotherapy-induced oral mucositis has been recognized as one of the key factors affecting the outcomes of the anticancer therapies. Therefore, the development of treatments mitigating oral mucositis would be of clinical significance, although the adequate assessment procedure for efficacy evaluation remains to be established. We conducted this post hoc study to assess the effect of objective evaluation of the severity grade on the outcomes of the clinical trial.

**Methods:**

In the original trial with rebamipide liquids (0, 2, and 4%) for chemoradiotherapy-induced oral mucositis, the investigators in local sites and independent central review separately determined the severity grades in accordance with Common Terminology Criteria of Adverse Events version 3.0 based on the Assessment Sheet scored by the investigators. The discordance in severity grades between the investigators and central review was analyzed on cross table.

**Results:**

The analysis revealed the discordance rate over the trial was 34%. While the incidences of severe oral mucositis in the placebo, rebamipide 2%, and 4% groups evaluated by the central review were 39%, 29%, and 25%, respectively, the respective values in the investigator’s evaluation were 32%, 39%, and 44%.

**Conclusion:**

In the clinical trial for the treatment of oral mucositis, it was strongly suggested that objective evaluation with a consistent scale would be required.

**Electronic supplementary material:**

The online version of this article (10.1007/s10147-018-1355-7) contains supplementary material, which is available to authorized users.

## Introduction

Mucositis, characterized as a mucosal injury on the gastrointestinal canal including oral cavity, can be caused secondarily by chemoradiotherapy (CRT) targeted on cancer [1]. The incidence of mucositis accompanied by anticancer therapy has been reported to be high, in almost 100% patients receiving head and neck radiation therapy [2–4]. Since some cases of mucositis are too severe to tolerate, its severity may adversely affect not only the quality of life of patients [5], but also the outcomes of the anticancer therapies [1]. For these reasons, effective therapies alleviating mucositis as the supportive care of cancers is a highly significant issue for cancer patients, in particular those with head and neck cancer (HNC) receiving CRT.

The disease severity of oral mucositis (OM) has been clinically assessed in accordance with the procedures aimed at evaluating the adverse events [6, 7], because OM was recognized as being caused by anticancer therapies. However, along with a surge of demand on the therapies for OM as mentioned above, a number of clinical trials aimed at developing prevention/treatment of OM have emerged recently [8]. While on establishing trial designs, the evaluation procedure has still several issues to resolve, such as which scale for OM grading is suitable for efficacy evaluation, or how frequently should periodic assessments be performed. Particularly, in multicenter trials for new treatments, procedures that minimize inter-assessor variability and ensure the optimal levels of consistency, accuracy, and precision are immediately required [8].

Based on mucus protective action of rebamipide [9–11], one of the treatments for gastritis and gastric ulcer [12], the phase 2 exploring trial had evaluated the preventive effect of rebamipide liquid on CRT-induced OM in patients with HNC, and the result demonstrated a potential availability of rebamipide against the incidence of severe OM [13].

In this original trial, the protocol had defined that investigators in each local site (INV) and the independent central review (ICR) [14] separately determined the severity grades of patients. Using the data mentioned above, we investigated the differences in severity grade scored between INV and ICR in the current post hoc analysis.

## Patients and methods

The aim of this post hoc analysis was to investigate the differences in grading between ICR and INV, and therefore the end point of this study was the comparison of oral mucositis evaluations between ICR and INV.

### Trial design, participants and treatment

The design of the original trial (a multicenter, randomized, double-blind, placebo-controlled, dose-ranging, phase 2 trial; ClinicalTrials.gov identifier: NCT02085460) has been already published in detail [13]. Briefly, patients with histopathological diagnosis of primary tumor scheduled to undergo definitive or postoperative CRT to head and/or neck were eligible for enrollment. Following confirmation of their eligibility, patients were randomly assigned to receive placebo, rebamipide 2%, or rebamipide 4% treatment in a 1:1:1 ratio. Treatment with the study drug started 3 days prior to CRT initiation and continued for another 77 days. The study drugs were given 6 times daily in accordance with the instruction to wash their mouths with 5 mL of the study drug and then swallow it. During the study, the treatment allocation code was kept at the randomization center, and the patients, investigators, study personnel belonging to the sponsor, clinical organizations, and the members of ICR were masked to treatment allocation.

### Oral assessment

**Fig. 1:**
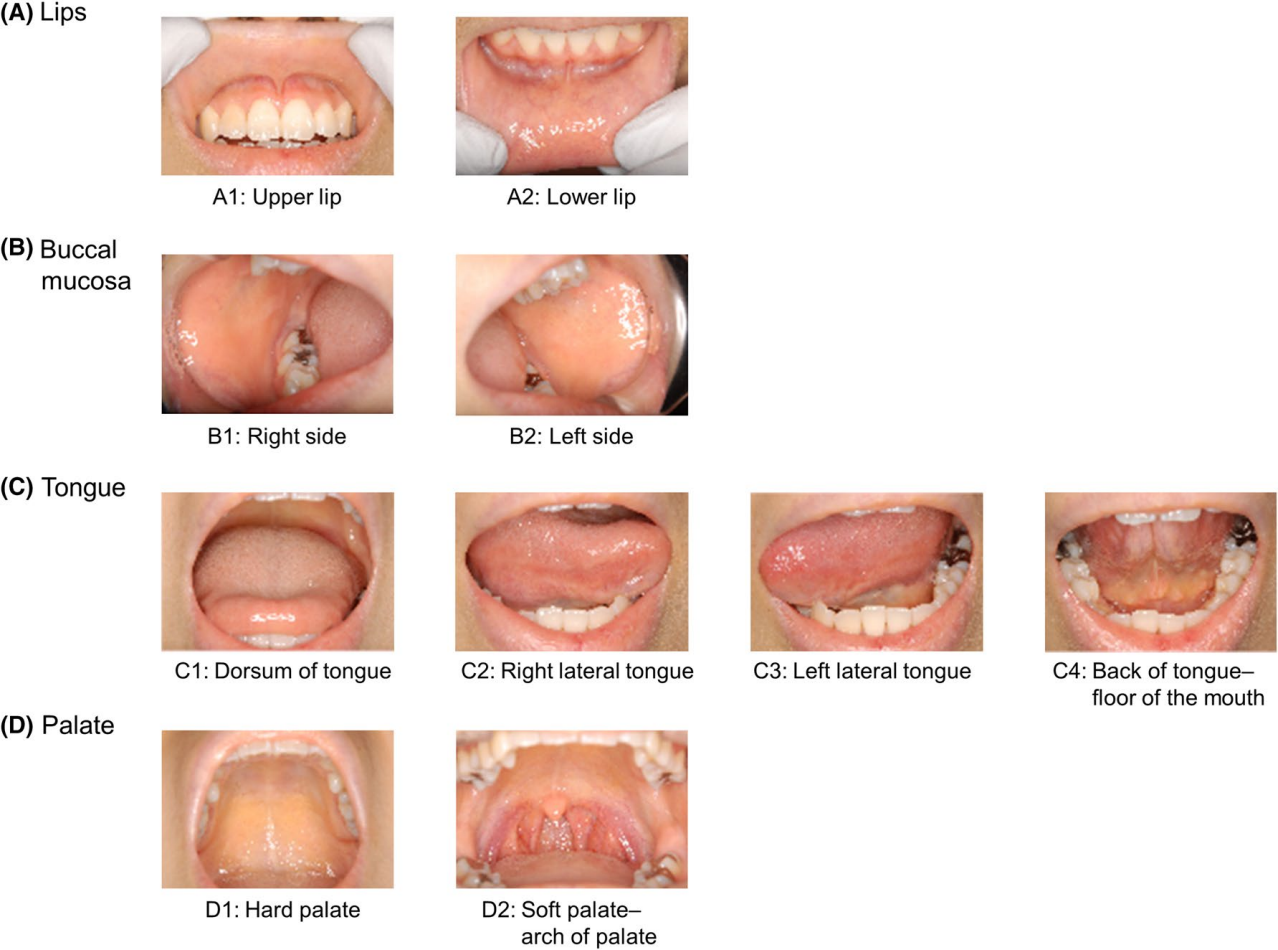
Pre-fixed 10 separate sites for evaluation of clinical examination in oral mucositis. On every evaluation day, the investigator at each local site observed all pre-fixed 10 separate sites (A1–D2) in the oral cavity of patients. They checked them with the score of “0”–“88” mentioned in “Oral Mucositis Assessment Sheet”, and filled the respective boxes

To evaluate the severity of OM, the CTCAE ver. 3.0 (Online Resource 1) and the Oral Mucositis Assessment Sheet (the Assessment Sheet: Online Resource 2) were employed. The ICR composed of four experts (Online Resource 3) had originally prepared the Oral Mucositis Assessment Sheet prior to the trial. The Assessment Sheet for the clinical examination consisted of a 10-ranged score (0–9) based on the condition of oral mucosa at pre-fixed 10 separate sites in the oral cavity (lips: A1: upper lip, A2: lower lip; buccal mucosa: B1: right side, B2: left side; tongue: C1: dorsum of tongue, C2: right lateral tongue, C3: left lateral tongue, C4: back of tongue–floor of the mouth; palate; D1: hard palate, D2: soft palate–arch of palate, Fig. 1), and that for the functional/symptomatic aspects comprised a 6-ranged score (0–5) based on the condition of oral nutritional intake.

INV who had undergone specific training for oral assessment before the trial, evaluated the severity of OM twice weekly. They recorded the clinical examination and functional/symptomatic aspects on the Assessment Sheet on every evaluation day. Then, based on their own Assessment Sheets, they determined the severity grade of OM in accordance with the CTCAE ver. 3.0. Thereafter, the Assessment Sheets were sent to the ICR to determine the severity of the OM separately.

“The severity grade in the evaluation opportunity” was defined as the maximum grade among the pre-fixed 10 sites in the oral cavity in the respective evaluation opportunities, and “the severity grade in patient” was defined as the maximum grade in all “the severity grades in the evaluation opportunities” in the treatment period of the respective patients.

### Statistical analysis

For the statistical analysis, the full analysis set (FAS) was utilized; placebo (*n* = 31), rebamipide 2% (*n* = 31), and rebamipide 4% (*n* = 32). We employed the incidence of severe OM in clinical examination determined by INV to compare with the incidence of severe OM in clinical examination determined by ICR, and determined the difference in evaluations scored between ICR and INV, the primary end point of the current post hoc study. The difference in the incidences of severe OM (grade ≥ 3) between the respective groups was evaluated through Chi square test [13].

For post hoc analysis, the comparison of the severity grades scored between the ICR and INV was performed on the cross table. The severity grades, which did not agree with each other, were defined as “discordance”. The number and grades of discordance were determined, and the number of discordances was divided by the number of patients to calculate the incidence of discordance (the discordance rate), one of the parameters for inter-assessor reliability.

The kappa (*κ*) statistics (not weighted), another parameter for inter-assessor reliability, was calculated according to the following equation [14]:

$$\kappa =({P_{\text{o}}} - {P_{\text{e}}})/(1 - {P_{\text{e}}}),$$

where *P*_o_ is the proportion of assessor pairs exhibiting concordance and *P*_e_ is the proportion expected to exhibit concordance by chance alone.

The standard error (SE) of *κ* was estimated with the following equation:

$${\text{SE}}=\sqrt {({P_{\text{o}}}[1 - {P_{\text{o}}}]/n{{[1 - {P_{\text{e}}}]}^2})} ,$$

where *n* is the total number of evaluations.

Finally, the 95% confidence interval (95% CI) was calculated as follows:

$$95\% \,{\text{CI}}=\kappa +1.96 \times {\text{SE}}.$$

Statistical analyses for the original and post hoc studies were conducted using Microsoft Office Excel 2007 and SAS version 9.2 or above (SAS Institute Inc, Cary, NC, USA), and JMP version 13 (SAS Institute Inc, Cary, NC, USA) for the post hoc analysis.

## Results

### Number of patients and patient characteristics

Of 97 patients randomized in 18 study sites, 94 patients (placebo: *n* = 31; rebamipide 2%: *n* = 31; rebamipide 4%: *n* = 32) received the study drugs and were the subjects of analysis.

As shown in Table 1, patients were allocated into the three treatment groups in a balanced manner. Equally among these treatment groups, the ratios of patients who had their primary tumor in the visible area of the oral cavity (e.g., oral cavity + oropharynx) and those who had previously undergone surgeries for HNC were approximately 60% and 20%, respectively.

**Table 1:** Patient baseline demographics

		Placebo (*N* = 31)	Rebamipide 2% (*N* = 31)	Rebamipide 4% (*N* = 32)
Gender: *n* (%)	Male	25 (81)	26 (84)	26 (81)
	Female	6 (19)	5 (16)	6 (19)
Age: year (mean ± SD)		60 ± 9	61 ± 12	62 ± 9
ECOG PS: *n* (%)	0	28 (90)	28 (90)	28 (88)
	1	3 (10)	3 (10)	4 (13)
Primary site: *n* (%)				
Oral cavity		2 (6)	4 (13)	4 (13)
Epipharynx		6 (19)	7 (23)	6 (19)
Oropharynx		17 (55)	14 (45)	15 (47)
Hypopharynx		5 (16)	6 (19)	6 (19)
Larynx		1 (3)	0 (0)	1 (3)
Prior surgery for head and neck cancer: *n* (%)	Yes	7 (23)	6 (19)	6 (19)
	No	24 (77)	26 (81)	26 (81)
Cancer stage: *n* (%)				
Stage I		1 (3)	1 (3)	0 (0)
Stage II		8 (26)	2 (7)	4 (13)
Stage III		5 (16)	6 (19)	7 (22)
Stage IV A		14 (45)	20 (65)	20 (63)
Stage IV B		3 (10)	2 (7)	1 (3)
Stage IV C		0 (0)	0 (0)	0 (0)
TMN staging of primary tumor: *n* (%)				
T1		6 (19)	9 (29)	4 (13)
T2		14 (45)	10 (32)	15 (47)
T3		7 (23)	4 (13)	5 (16)
T4		4 (13)	8 (26)	8 (25)
N0		7 (23)	3 (10)	3 (9)
N1		8 (26)	3 (10)	8 (25)
N2		13 (42)	23 (74)	20 (63)
N3		3 (10)	2 (6)	1 (3)
Radiation technique: *n* (%)				
3D-CRT		4 (13)	9 (29)	7 (22)
IMRT		27 (87)	22 (71)	25 (78)

Data are presented as number and percent: *n* (%)

*SD* standard deviation, *ECOG PS* Eastern Cooperative Oncology Group performance status, *3D-CRT* three-dimensional conformal radiation therapy, *IMRT* intensity-modulated radiation therapy

### Comparison of the incidences of severe mucositis scored between the ICR and INV

**Fig. 2:**
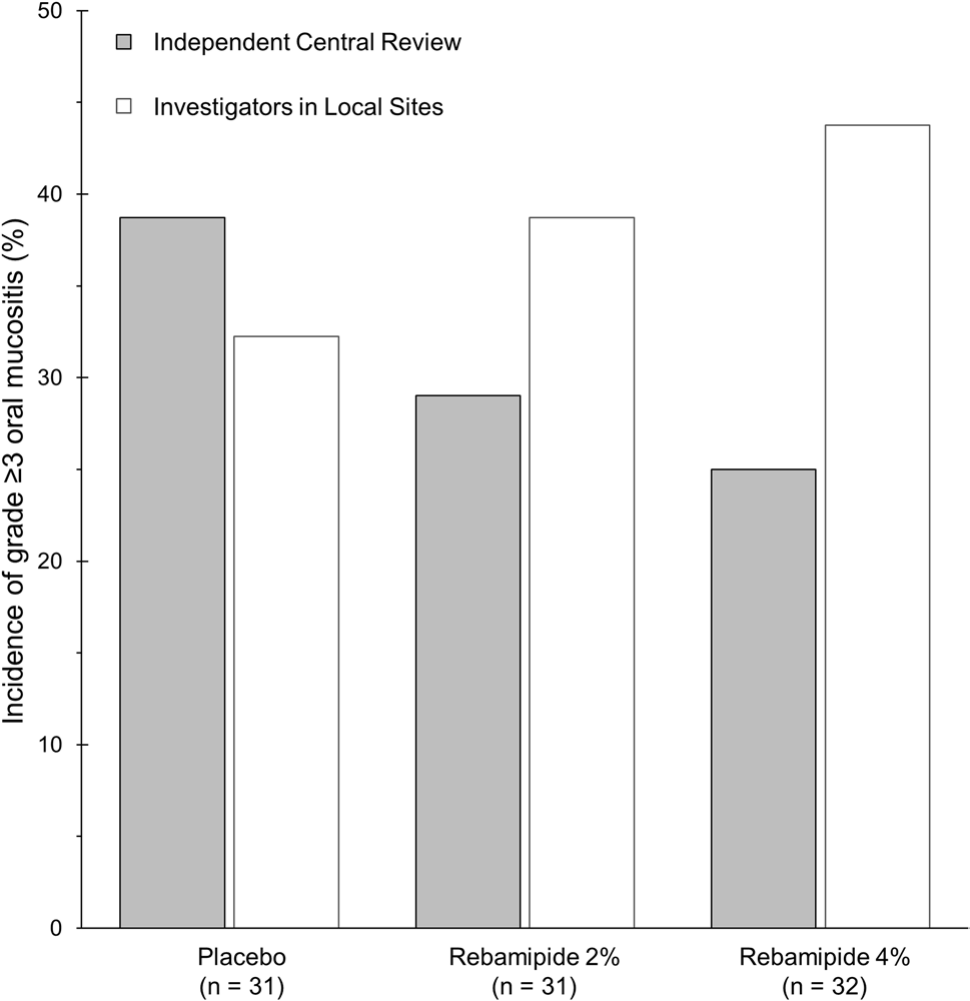
Comparison of the severity grades of clinical examination. The graph demonstrates the incidence of grade ≥ 3 oral mucositis based on clinical examination evaluated by the independent central review (shaded columns) or the investigators in local sites (open columns)

The incidence of severe mucositis (grade ≥ 3) in clinical examination determined by the ICR was compared with that by INV (Fig. 2). The incidences of severe mucositis in the placebo, rebamipide 2%, and 4% groups in the ICR grading were 39% (*n* = 31), 29% (*n* = 31, *p* = 0.421 vs. placebo), and 25% (*n* = 32, *p* = 0.243 vs. placebo), respectively. On the contrary, those in the INV grading were 32%, 39% (*p* = 0.596 vs, placebo), and 44% (*p* = 0.348 vs. placebo), respectively.

Table 2 demonstrates the comparison of severity grades over the trial scored between the ICR and INV in the cross table. This comparison revealed that the number and the incidence of discordance were 32 cases and 34%, respectively. Furthermore, except for a single case (ICR grade: 4 and INV grade: 2) with a difference by two grades, the differences in grade of other discordances were one, and the highest incidence of discordance was observed at ICR grade 2 and INV grade 3 with 14 cases. The *κ* statistics, the proportion of concordance beyond that expected by chance, was calculated in not weighted form as 0.49 (SE 0.075; 95% CI 0.34–0.64).

**Table 2:**
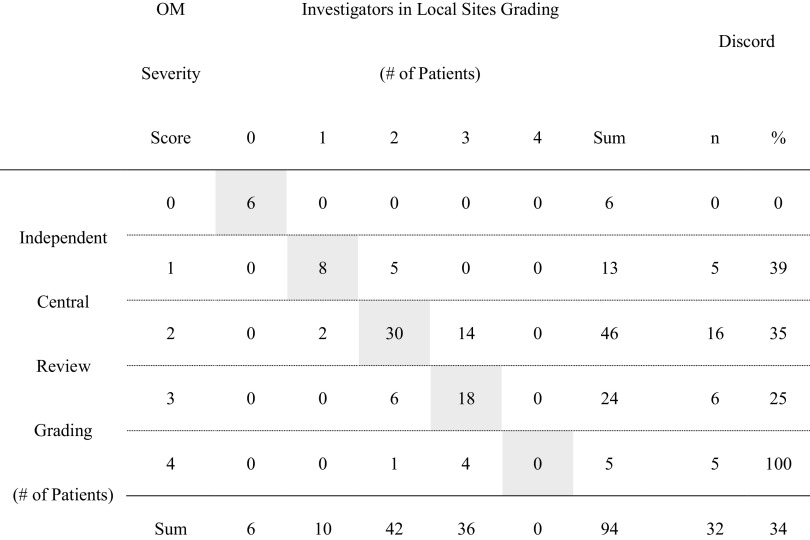
Comparison of oral mucositis evaluations between the independent central review and investigators at local sites: patient-by-patient analysis—all patients

Each cell of the cross table was filled with the number of patients according to the respective severity which was evaluated by the independent central review and investigators at local sites. Hence, the number on the diagonal (the shaded cells) demonstrates that of the patients whose severity scores determined by the investigators are equal to those by the committee, the evaluations in the area other than the diagonal are different between the central review and investigators. In addition, the number in the “%” columns in “Concordant” and “Discordant” express the ratio to the respective number in the “Sum” column in “Investigators in Local Sites Grading”

*OM* oral mucositis

**Fig. 3:**
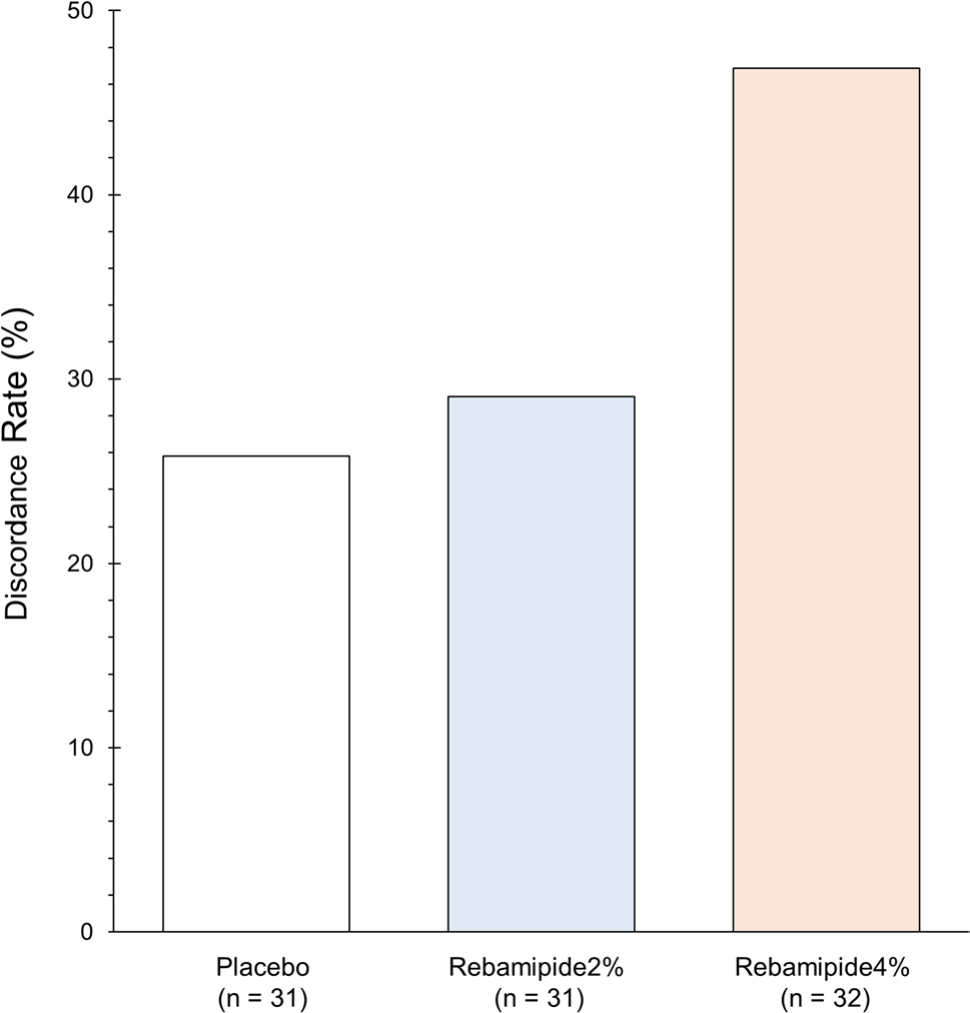
The discordance rates in the respective dosing groups. The discordance number of the respective dosing groups was divided by the respective number of patients

Figure 3 demonstrates the discordance in the respective treatment groups. The numbers of discordance (the discordance rates) of the placebo, rebamipide 2%, and rebamipide 4% groups were 8/31 (26%), 9/31 (29%) and 15/32 (47%), respectively.

## Discussion

The present post hoc study was performed to compare the severity grades in CRT-OM scored between the ICR and INV, and to assess the effect of the differences on the outcomes of the rebamipide phase 2 trial in patients with HNC. The result revealed that a discordance rate in severity grades at the patient level was 34%, and the opposite outcomes on treatment efficacies were obtained (the incidences of severe OM decreased with doses of rebamipide in the ICR, but increased in INV).

To our knowledge, this study is the first attempt to investigate the differences in severity grade in CRT-OM scored between the ICR and INV in a prospective double-blind, intervention clinical trial. Therefore, the outcomes in the present study could not be compared with those of others. So, we retrieved the articles in the areas beyond CRT-OM in patients with HNC, and we could find several. All of them compared the outcomes scored between ICR and INV in clinical trials for anti-cancer drugs and indicated that there was a consistent trend in which the response rates of the ICR were always lower than those of INV [15–18]. In addition, some of them also reported the discordance rates at patient level to be over 40% [15–17]. In comparison with them, the response rates scored by the ICR in the present study were not lower than those by INV, and the discordance rate at patient level in the current study (34%) was lower. The differences in the outcomes observed between this study and others may result from differences in the target diseases (cancer vs. oral mucositis), the evaluation methods (radiological review vs. visual judgment) and the proficiency levels for evaluation of INV. However, unfortunately we do not have reliable answers on it at this moment.

In addition to the discordance rate, we calculated *κ* statistic to evaluate the inter-assessor reliability of the current study. An index *κ* statistic, a parameter of concordance rate corrected for chance, is often employed to evaluate inter-assessor reliability. We found that the *κ* statistic of the current study was 0.49, and this value would be evaluated as “moderate (0.41–0.60)” in accordance with the interpretation of Landis and Koch [20]. McHugh advocates values of 0.40–0.59 as “weak”, because a clinical facility having 40% of incorrect evaluations would be deemed to have critical issue in terms of quality [21]. Given the effect on the outcome of the original trial (Fig. 2), we concluded it as “serious”.

It was supposed that the “datasets” used for review and the “review process” which had been reported to cause the discordance between the ICR and INV [14], might play a key role in the current study. Firstly, the “datasets” disclosed to the assessors had a large difference between the ICR and INV. On the evaluation by INV performed as a part of clinical care, much information other than the lesion of OM, such as the state of primary cancers in the oral cavity or complaints from patients, would be provided to the assessors regardless of whether they wanted it or not. On the other hand, the members of the ICR were provided just information of the lesion of OM, and blinded to the various biasing information of patients. Given these differences in datasets, it was suggested that the “subjective assessment of non-target disease” would likely affect the evaluation by INV. In addition, there were also distinct differences in the “review process” between the ICR and INV. The evaluation scores provided from the ICR were warranted to be highly consistent, because a small number of experts (four physicians) performed the severity grading to examine the efficacy of the treatments. Although specific training had been performed for INV aimed at lowering the “variability in protocol training” prior to the trial, the discordance rate was high. This fact implies that more in-depth training for INV aimed at standardization of the evaluation including rigorous photo finish would be necessary to reduce the discrepancy.

There is a limitation in the current study. The comparison of severity grade in functional/symptomatic aspects was not performed due to insufficient effect of rebamipide on functional/symptomatic aspects [13]. For this reason, future comparison of evaluation on functional/symptomatic aspects in OM will be designed for the study drugs, which have actions against dysgeusia, salivary gland secretion or swallowing dysfunction. Furthermore, from a perspective of supportive care in cancer, it would be required to measure patient symptoms such as pain, fatigue, or emotional distress, in addition to clinical manifestation determined by clinicians’ objective evaluation in clinical trials for the treatment of CRT-OM. To achieve such purposes, validated self-reported health-measuring systems, such as Oral Mucositis Daily Questionnaire or Patient-Reported Oral Mucositis Symptom should be employed.

Taken together, it is supposed that discordance in evaluation between the ICR and INV would be inevitable. Although the physician and the patient should make the final decision on therapeutic measures in clinical care, it is strongly suggested that an objective evaluation with a consistent scale would be required in clinical trials in the treatment for CRT-OM for patients with HNC to reduce the discordance as much as possible.

## Electronic supplementary material

Below is the link to the electronic supplementary material.

Supplementary material 1 (PDF 289 KB)

Supplementary material 2 (PDF 371 KB)

Supplementary material 3 (PDF 211 KB)
