# Supplementary material for: The post hoc analysis comparing the severity grades of chemoradiotherapy-induced oral mucositis scored between the central and local assessors in a multicenter, randomized controlled trial of rebamipide for head and neck cancer
Source: Int J Clin Oncol. 2018 Nov 13;24(3):241–7. doi: 10.1007/s10147-018-1355-7 (PMC6399175; doi:10.1007/s10147-018-1355-7)
Supplement: Supplementary file 1 — Supplementary material 1 (PDF 289 KB) [file 10147_2018_1355_MOESM1_ESM.pdf]

Electronic Supplementary Material_1

Title: The post-hoc analysis comparing the severity grades of chemoradiotherapy-induced oral mucositis scored between the central and local assessors in a multicenter, randomized controlled trial of rebamipide for head and neck cancer

Journal Name: International Journal of Clinical Oncology

Authors: Takao Ueno, Sadamoto Zenda, Tetsuhito Konishi, Takashi Yurikusa, Yoshiyuki Shibasaki, Hisashi Nagamoto, Masato Fujii

Corresponding Author:

Name: Sadamoto Zenda

Affiliation: Division of Radiation Oncology and Particle Therapy, National Cancer Center Hospital East, 6-5-1 Kashiwa-no-ha, Kashiwa, Chiba, 277-8577, Japan

e-mail address: szenda@east.ncc.go.jp

Online Resource 1. Common Terminology Criteria for Adverse Events version 3.0 (CTCAE ver. 3.0)

	Grade 1	Grade 2	Grade 3	Grade 4	Grade 5
Mucositis (Clinical Examination)	Erythema of the mucosa	Patchy ulcerations or pseudomembranes	Confluent ulcerations or pseudo-membranes; bleeding with minor trauma	Tissue necrosis; significant spontaneous bleeding; life-threatening consequences	Death
Mucositis (Functional/ Symptomatic Aspects)	Minimal symptoms, normal diet;	Symptomatic but can eat and swallow modified diet;	Symptomatic and unable to adequately aliment or hydrate orally;	Symptoms associated with life-threatening consequences	Death
