# Supplementary material for: The post hoc analysis comparing the severity grades of chemoradiotherapy-induced oral mucositis scored between the central and local assessors in a multicenter, randomized controlled trial of rebamipide for head and neck cancer
Source: Int J Clin Oncol. 2018 Nov 13;24(3):241–7. doi: 10.1007/s10147-018-1355-7 (PMC6399175; doi:10.1007/s10147-018-1355-7)
Supplement: Supplementary file 2 — Supplementary material 2 (PDF 371 KB) [file 10147_2018_1355_MOESM2_ESM.pdf]

Electronic Supplementary Material_2

Title: The post-hoc analysis comparing the severity grades of chemoradiotherapy-induced oral mucositis scored between the central and local assessors in a multicenter, randomized controlled trial of rebamipide for head and neck cancer

Journal Name: International Journal of Clinical Oncology

Authors: Takao Ueno, Sadamoto Zenda, Tetsuhito Konishi, Takashi Yurikusa, Yoshiyuki Shibasaki, Hisashi Nagamoto, Masato Fujii

Corresponding Author:

Name: Sadamoto Zenda

Affiliation: Division of Radiation Oncology and Particle Therapy, National Cancer Center Hospital East, 6-5-1 Kashiwa-no-ha, Kashiwa, Chiba, 277-8577, Japan

e-mail address: szenda@east.ncc.go.jp

Online Resource 2. Oral Mucositis Assessment Sheet

Oral mucositis assessment sheet

Subject No.: -

Date of assessment: Month/date/year

Assessor:

Observation			No change in the mucosa	Redness (erythema)	Color changes in the mucosa to white or pallor	Mucosal edema	Detachment of the epithelium from small ulcerative lesions (sporadic and non-contiguous)	White pseudo-membrane covering the surface	Widespread ulcerative lesions over subsites by >30 mm	Easy bleeding from ulcerative lesions with contact	Spontaneous bleeding from ulcerative lesions	Necrosis of ulcerative surface	Mucosa not evaluable (flaps, etc.)
			0	1	2	3	4	5	6	7	8	9	88
Lips	Upper lip	A1	☐	☐	☐	☐	☐	☐	☐	☐	☐	☐	☐
	Lower lip	A2	☐	☐	☐	☐	☐	☐	☐	☐	☐	☐	☐
Buccal mucosa	Right side	B1	☐	☐	☐	☐	☐	☐	☐	☐	☐	☐	☐
	Left side	B2	☐	☐	☐	☐	☐	☐	☐	☐	☐	☐	☐
Tongue	Dorsum of tongue	C1	☐	☐	☐	☐	☐	☐	☐	☐	☐	☐	☐
	Right lateral tongue	C2	☐	☐	☐	☐	☐	☐	☐	☐	☐	☐	☐
	Left lateral tongue	C3	☐	☐	☐	☐	☐	☐	☐	☐	☐	☐	☐
	Back of tongue-floor of the mouth	C4	☐	☐	☐	☐	☐	☐	☐	☐	☐	☐	☐
Palate	Hard palate	D1	☐	☐	☐	☐	☐	☐	☐	☐	☐	☐	☐
	Soft palate-arch of palate	D2	☐	☐	☐	☐	☐	☐	☐	☐	☐	☐	☐

Oral intake status	E	☐[0] Good oral intake: normal food (the same texture as that for the family) intake is maintained.
		☐[1] Oral intake is feasible with slight modifications in seasoning and texture of food (soft food, etc.)
		☐ The proportion of rice porridge, liquid diet and dietary supplements is ≥50%.
		☐[2] The main causes are nausea and dysgeusia.
		☐[3] The main causes are pain in the oral cavity (mucositis, etc.).
		☐ Inability to adequately aliment orally (gastric fistula, tube feeding)
		☐[4] The main causes are nausea and dysgeusia
		☐[5] The main causes are pain in the oral cavity (mucositis, etc.).

*Check boxes that apply.
