# Supplementary material for: The post hoc analysis comparing the severity grades of chemoradiotherapy-induced oral mucositis scored between the central and local assessors in a multicenter, randomized controlled trial of rebamipide for head and neck cancer
Source: Int J Clin Oncol. 2018 Nov 13;24(3):241–7. doi: 10.1007/s10147-018-1355-7 (PMC6399175; doi:10.1007/s10147-018-1355-7)
Supplement: Supplementary file 3 — Supplementary material 3 (PDF 211 KB) [file 10147_2018_1355_MOESM3_ESM.pdf]

Electronic Supplementary Material_3

Title: The post-hoc analysis comparing the severity grades of chemoradiotherapy-induced oral mucositis scored between the central and local assessors in a multicenter, randomized controlled trial of rebamipide for head and neck cancer

Journal Name: International Journal of Clinical Oncology

Authors: Takao Ueno, Sadamoto Zenda, Tetsuhito Konishi, Takashi Yurikusa, Yoshiyuki Shibasaki, Hisashi Nagamoto, Masato Fujii

Corresponding Author:

Name: Sadamoto Zenda

Affiliation: Division of Radiation Oncology and Particle Therapy, National Cancer Center Hospital East, 6-5-1 Kashiwa-no-ha, Kashiwa, Chiba, 277-8577, Japan

e-mail address: szenda@east.ncc.go.jp

Online Resource 3. Members of the Oral Mucositis Evaluation Committee

Name	Facility
Takao Ueno, MD, Ph.D.	Department of General Internal Medicine, Dentistry, Oncologic Emergency, National Cancer Center Hospital, Tokyo, Japan
Sadamoto Zenda, MD, Ph.D.	Division of Radiation Oncology and Particle Therapy, National Cancer Center Hospital East, Chiba, Japan
Tetsuhito Konishi, MD, Ph.D.	Department of Dentistry, National Cancer Center Hospital East, Chiba, Japan
Takashi Yurikusa, MD, Ph.D.	Division of Dentistry and Oral Surgery, Shizuoka Cancer Center, Shizuoka, Japan
